# Leaf economics spectrum–productivity relationships in intensively grazed pastures depend on dominant species identity

**DOI:** 10.1002/ece3.1964

**Published:** 2016-04-02

**Authors:** Norman W.H. Mason, Kate Orwin, Suzanne Lambie, Sharon L. Woodward, Tiffany McCready, Paul Mudge

**Affiliations:** ^1^ Landcare ResearchPrivate Bag 3127Hamilton3240New Zealand; ^2^ Landcare ResearchPO Box 40Lincoln7640New Zealand; ^3^ DairyNZPrivate Bag 3221Hamilton3240New Zealand; ^4^ Present address: 27 Delaware CresChristchurchNew Zealand; ^5^ Present address: Seed Force New Zealand LtdPO Box 16 625Christchurch8441New Zealand

**Keywords:** Agriculture, biodiversity, biodiversity–ecosystem function relationships, dairy farming, ecosystem function, intensively managed pastures, niche complementarity, over‐yielding, pasture

## Abstract

Plant functional traits are thought to drive variation in primary productivity. However, there is a lack of work examining how dominant species identity affects trait–productivity relationships. The productivity of 12 pasture mixtures was determined in a 3‐year field experiment. The mixtures were based on either the winter‐active ryegrass (*Lolium perenne*) or winter‐dormant tall fescue (*Festuca arundinacea*). Different mixtures were obtained by adding forb, legume, and grass species that differ in key leaf economics spectrum (LES) traits to the basic two‐species dominant grass–white clover (*Trifolium repens*) mixtures. We tested for correlations between community‐weighted mean (CWM) trait values, functional diversity, and productivity across all plots and within those based on either ryegrass or tall fescue. The winter‐dormant forb species (chicory and plantain) had leaf traits consistent with high relative growth rates both per unit leaf area (high leaf thickness) and per unit leaf dry weight (low leaf dry matter content). Together, the two forb species achieved reasonable abundance when grown with either base grass (means of 36% and 53% of total biomass, respectively, with ryegrass tall fescue), but they competed much more strongly with tall fescue than with ryegrass. Consequently, they had a net negative impact on productivity when grown with tall fescue, and a net positive effect when grown with ryegrass. Strongly significant relationships between productivity and CWM values for LES traits were observed across ryegrass‐based mixtures, but not across tall fescue‐based mixtures. Functional diversity did not have a significant positive effect on productivity for any of the traits. The results show dominant species identity can strongly modify trait–productivity relationships in intensively grazed pastures. This was due to differences in the intensity of competition between dominant species and additional species, suggesting that resource‐use complementarity is a necessary prerequisite for trait–productivity relationships.

## Introduction

There is a growing literature linking plant functional traits to primary productivity (Garnier et al. 2004; Mouillot et al. 2011; Clark et al. 2012; Roscher et al. 2012). However, much of this work has focussed on ecosystems, such as low‐intensity semi‐natural grasslands, where maximizing productivity is unlikely to be the primary aim of management (Lepš 2004). Comparatively little work has been carried out on intensive pastoral systems where increasing production with fewer external inputs is the main concern of managers (but see Finn et al. 2013). Trait–productivity relationships may reveal processes behind variation in productivity between different pastoral mixtures and help design new mixtures for increased productivity (e.g., Storkey et al. 2015). However, it is still not known if trait–productivity relationships emerging from the literature are truly general or highly dependent on species identity, environmental context, and management practices. There also appears to be a lack of work examining how interactions between different trait axes (e.g., the leaf economics spectrum – LES – and seasonal growth phenology) alter trait–productivity relationships. This study tests whether relationships between leaf morphological traits and productivity in intensively managed pasture mixtures are influenced by dominant species identity.

### Trait composition and complementarity as predictors of ecosystem function

The mass ratio hypothesis predicts that a species influence on ecosystem function is proportionate to its abundance (Grime 1998). This has been extended to predict that ecosystem function should depend on the traits of the dominant species (Vile et al. 2006). This in turn has led to a large body of research testing how well the community‐weighted mean (CWM) of basic plant traits predicts key ecosystem functions such as primary productivity and litter decomposition (e.g., Quested et al. 2007; Mokany et al. 2008). Complementarity (Loreau and Hector 2001) is the other major hypothesis linking traits to ecosystem function. Here, trait differences are hypothesized to enhance ecosystem function by increasing total community resource acquisition through spatial and temporal resource partitioning between species (Tilman et al. 1997; Petchey et al. 2004). This has led to a number of studies testing how well functional group richness or functional diversity indices predict ecosystem function. Some attempts have been made to determine which hypothesis has the greater influence on ecosystem function (Mokany et al. 2008; Roscher et al. 2012), but like many such debates in ecology, both are likely to have some influence (Mouillot et al. 2011), with their relative importance varying according to the context.

### Leaf economics spectrum, resource‐use differentiation, and productivity

Extensive research on the leaf economics spectrum (LES; Wright et al. 2004) has demonstrated that basic morphological traits (e.g., specific leaf area – SLA; leaf tissue density or its proxy leaf dry matter content – LDMC; and leaf thickness) are strongly linked to maximum leaf‐level photosynthesis (e.g., Niinemets 1999; Reich et al. 1999). LDMC and leaf thickness are often negatively correlated with SLA, but they influence photosynthesis in different ways (Niinemets 1999). Maximum photosynthesis per unit leaf area increases with leaf thickness and decreases with SLA, while photosynthesis per unit leaf dry weight increases with SLA and decreases with LDMC (Niinemets 1999). SLA and net assimilation rate (NAR – growth rate per unit leaf area) both strongly determine maximum whole‐plant relative growth rate (RGR; Shipley 2006), suggesting that photosynthesis on both a per unit area and a per unit dry‐weight basis are likely to be important for RGR.

SLA, LDMC, and RGR of the dominant species can be strong predictors of ecosystem‐level primary productivity (e.g., Garnier et al. 2004; Vile et al. 2006). Thus, communities dominated by species with LES traits linked to high RGR should have the highest levels of productivity under conditions of high resource availability (Vile et al. 2006). However, some studies have found only weak or even nonsignificant relationships between community‐weighted mean (CWM) values for LES traits and productivity (e.g., Mokany et al. 2008; Mouillot et al. 2011). Thus, it seems that dominance by species with traits linked to high RGR does not guarantee high ecosystem‐level productivity.

There is building evidence that complementarity in resource acquisition traits (i.e., indicators of when and where species obtain their resources from) can reduce interspecific competition and enhance productivity. For instance, Roscher et al. (2011) found that functional diversity of delta N^15^ signatures (an indicator of nitrogen acquisition strategy) significantly improved models predicting productivity, while Fargione and Tilman (2005) showed that differences in rooting depth can reduce interspecific competition. Complementarity in these traits is thought to reduce competition by allowing differentiation in belowground resource use (Fargione and Tilman 2005), which enhances productivity by increasing total community resource use. There is also evidence that phenological complementarity enhances productivity (Stevens and Carson 2001; Mouillot et al. 2011) and may promote species coexistence (Fargione and Tilman 2005; Mason et al. 2013). Phenological complementarity can both reduce competition between species by allowing temporal differentiation in resource use (Fargione and Tilman 2005) and enhance productivity by increasing total annual resource use (Stevens and Carson 2001).

As LES traits are so tightly linked to RGR (Shipley 2006) and the RGR of dominant species is often such a strong determinant of ecosystem‐level productivity (Vile et al. 2006), we should expect LES traits to strongly influence productivity via the mass ratio hypothesis. In contrast, any influence of resource acquisition traits, independent of LES traits, is likely to be via complementarity. Complementarity in resource acquisition traits could alter relationships between LES traits and productivity, through their influence on the intensity of interspecific competition. In the absence of complementarity in resource acquisition, intense competition between high RGR species and other species may reduce the positive effects on productivity of adding high RGR species to a mixture. However, no work has been carried out examining whether the influence of LES traits on productivity is altered by complementarity in resource acquisition traits.

### Aims and objectives

Here, the productivity of 12 pasture mixtures has been compared in a 3‐year field experiment. The mixtures were based on one of two grasses (perennial ryegrass *Lolium perenne* and tall fescue *Festuca arundinaceae* – termed base grasses for simplicity) with differing levels of winter dormancy (Malcolm et al. 2014); different mixtures were obtained by altering the other species grown with these grasses. The additional species used differ markedly in key LES traits, providing a wide range of CWM values across mixtures based on either of the grass species. This provides an excellent opportunity to test whether the influence of LES traits on productivity is contingent on dominant species identity. In particular, we test the hypothesis that adding species with traits linked to high RGR to pasture mixtures will only increase productivity when they differ in phenology from the base grass.

## Methods

### Study area

The experiment was located on DairyNZ's Scott Farm, near Hamilton in the North Island, New Zealand (37°46′16″S, 175°21′39″E). The mean annual temperature is 13.6⁰C with a mean annual rainfall of 1224 mm. Winters are relatively mild (mean temperature in the coldest month is 4.2⁰C), and water deficit in summer and autumn is moderate to high (mean deficit – estimated using the Penman–Monteith equation for potential evapotranspiration – during summer and autumn was 71 ± 21 mm for the 3 years of the study). The soil at the experimental site is the Matangi silt loam (Typic Orthic Gley Soil; Hewitt 1993). Typical annual dry matter production for pastures at Scott Farm is 15–22 t·ha^−1^·year^−1^ with an average of 19 t·ha^−1^·year^−1^ (Glassey et al. 2013).

### Experimental design

In March 2010, 12 different pasture mixtures were sown in 9 × 6 m plots (using a roller drill), following spraying with herbicide (glyphosate‐based) to kill the existing sward, mouldboard plowing, and power harrowing. Six mixtures were based on perennial ryegrass (Lolium perenne L. ‘One50’ inoculated with the AR1 endophyte), and six were based on tall fescue (*Festuca arundinacea* ‘Advance' inoculated with the Max P endophyte). The ryegrass‐ and tall fescue‐based mixtures each followed the same series of species additions. Three replicates of each mixture were sown in a randomized block design (see Fig. S1 in the supplementary material for a schematic map of the experimental design). The “standard” mixtures included white clover (*Trifolium repens* ‘Kopu2') with either ryegrass or tall fescue (mixture codes RGST and TFST, respectively). There were two “legume” treatments where either of two legume species was added to the standard mixtures: red clover (*Trifolium pratense* ‘Colenso' – mixture codes RGLA and TFLA) or lucerne (*Medicago sativa* ‘Torlesse' – RGLB and TFLB). The “grasses” treatments added prairie grass (*Bromus willdenowii* ‘Atom') and timothy (*Phleum pratense* ‘Charlton') to the standard mixtures (mixture codes RGGR and TFGR). The forbs treatments added narrow‐leaved plantain (*Plantago lanceolata* ‘Tonic') and chicory (*Chicorium intybus* ‘Choice') to the standard mixtures (mixture codes RGFB and TFFB). Finally, the complex treatments added all additional legume, grass and forb species to the standard mixtures (mixture codes RGCO and TFCO). The sown species composition of the ryegrass‐ and tall fescue‐based mixtures is summarized in Table 1, and seed sowing rates for each species in each mixture are given in Table S1.

**Table 1:** Species sown in each of the 12 pasture treatments. Mixture codes beginning with RG indicate that perennial ryegrass was sown as the base grass. Those beginning with TF indicate that tall fescue was the base grass

	Ryegrass set		Tall fescue set	
Standards	RGST	Perennial ryegrass White Clover	TFST	Tall Fescue White Clover
Standards + legumes A	RGLA	Perennial ryegrass White Clover Red clover	TFLA	Tall Fescue White Clover Red clover
Standards + legumes B	RGLB	Perennial ryegrass White Clover Lucerne	TFLB	Tall Fescue White Clover Lucerne
Standards + forbs	RGFB	Perennial ryegrass White Clover Chicory Plantain	TFFB	Tall Fescue White Clover Chicory Plantain
Standards + grasses	RGGR	Perennial ryegrass White Clover Prairie Grass Timothy	TFGR	Tall fescue White Clover Prairie Grass Timothy
Complex	RGCO	Perennial ryegrass White Clover Prairie Grass Timothy Red clover Lucerne Chicory Plantain	TFCO	Tall fescue White Clover Prairie Grass Timothy Red clover Lucerne Chicory Plantain

### Plot management

Management of the plots was designed to replicate, as much as possible, conventional dairy pasture management. Plots were grazed 10–12 times each year using 2–3 cows per plot (depending on estimated feed) for 2–3 h. Cows were removed once residual feed was reduced to approximately 1500–1700 kg dry matter·(DM) ha^−1^. Occasionally in spring and summer, small portions of plots were not fully grazed to the desired residual biomass. These areas were mown with a lawnmower at its highest setting of 10 cm to remove seed heads and increase uniformity in residual biomass (such ‘topping’ is common practice in dairy pasture management in New Zealand).

### Yield and botanical data collection

The day before grazing, forage yield and botanical composition were estimated by harvesting a 0.85 × 5 m strip using a Jenquip^™^ (Jenquip, Fielding, New Zealand) harvester. The cutting height of the harvester was set to 4 cm, which represents the optimum height of dairy cow grazing. Harvests were taken sequentially from the center of one of three 6 × 1 m evenly spaced strips, so that consecutive harvests were always taken from a different strip. Thus, each strip was harvested every third grazing event, with a minimum harvest return time of 6 weeks for a single strip during peak growth, when grazing events occur every 2 weeks. The fresh weight of the harvested herbage was measured to the nearest 0.05 kg in the field. From this, a representative 1 kg sample was taken for dry weight measurement and sorting into species.

This sample was mixed thoroughly in the laboratory, with any large pieces of herbage cut to a size that was representative of the rest of the sample. From the 1 kg sample, three 200 g subsamples were taken for the estimation of the fresh weight to dry weight ratio. This ratio was multiplied by the harvest fresh weight to estimate the dry weight yield of the harvest. A subsample was then taken for sorting to species level. The weight of the subsample depended on leaf size and the complexity of the mixture sampled, with larger subsamples taken in mixtures containing more species and in those dominated by large fragments. In general, enough fragments were taken to provide a representative sample of the mixture (usually around 400). In instances where harvested biomass was dominated by small fragments, enough material was taken to obtain a minimum sample weight of 30 g. The dry weight of each species was measured, and the percentage contribution of each species to total dry weight was calculated. Mean daily forage dry matter production (kg·ha^−1^·day^−1^, for brevity referred to as productivity henceforth) was calculated through division of estimated dry weight by the number of days since the previous harvest. Mean productivity across harvests (weighted by the time since previous harvest) was calculated for each plot, thus providing a single productivity value for each plot covering the all three years of the experiment.

### Trait data collection

Morphological trait data were collected in October 2013. Material for trait measurement was collected at this time of year because this is a period of very high productivity when the plants are expected to be close to their maximum growth rates. Material for trait measurements was collected from the ryegrass and tall fescue standard mixtures (RGST and TFST) as well as the two complex mixtures (RGCO and TFCO) to allow for potential trait plasticity effects (there were no significant intraspecific differences between mixtures). In each plot where material was collected, five 0.5 × 0.5 m quadrats were randomly located. For each species sown in the plot, the two uppermost fully expanded leaves were taken from the two largest individuals within the quadrat. For species not occurring in the quadrat, leaves were collected from the two largest individuals within a 1 m radius of the quadrat.

Leaf collection took place early in the morning during a period of high soil moisture availability and low evaporative load, to ensure leaves were harvested close to maximum water content. Collected leaves were placed immediately between damp paper towels in agreement with standard protocol (Perez‐Harguindeguy et al. 2013), and once collections were completed for each plot, leaves were placed in a sealed plastic bag that was then placed in an icebox to avoid water loss. In the laboratory, petioles were removed from leaves of the two forb species and petiolules were removed from leaflets of the three legume species. Fresh weights for leaf dry matter content (LDMC) were measured as soon as field collections were finished. Leaf (or leaflet) maximum length and width were measured to the nearest millimeter using a ruler, and leaf thickness was measured to the nearest 10th of a millimeter using a micrometer. For the leaves of the two forb species and the leaflets of the legume species, length and width were entered into the equation for an ellipse to obtain an estimate of leaf area. For the leaf blades of grasses, the standard equation for the area of a triangle was used (as the maximum width always occurred at the base of the leaf blade).

### Functional composition and diversity

Functional composition and diversity for each harvest were estimated using the community‐weighted mean value (CWM) and functional divergence (i.e., the degree of functional difference between the most abundant species, FDiv, Villeger et al. 2008) of each of the LES traits measured (SLA, LDMC, and leaf thickness). FDiv was chosen as the measure of functional diversity because it is thought to be a reliable indicator of niche complementarity (Mouchet et al. 2010), and has been shown to be a strong predictor of productivity in biodiversity–ecosystem function experiments (Mouillot et al. 2011) and was not strongly correlated with CWM for any of the traits. Harvest biomass measurements were used as species abundance weights. For each plot, the CWM values were averaged for each trait across harvests, thus obtaining a single value for each trait, for each plot. CWM values for each harvest were weighted by the length of time since the previous harvest, so that the contribution of each harvest to the plot mean was proportional to the length of time its CWM values covered.

### Seasonal growth phenology

Differences in species seasonal growth patterns were determined using an index of “spring growth response”. This index expresses the difference between the spring (defined as the 1st of September to the 30th of November) and winter (the 1st of June to the 31st of August) productivity of each species as a proportion of winter productivity. This was calculated separately for each year in each plot where a species was sown. Productivity for individual species was estimated as the product of the species proportional abundance in harvests and total plot productivity. This measure was chosen as the indicator of species phenology because it is robust against interannual weather fluctuations, particularly in water deficit. The productivity of all species used is known to increase reliably with increases in day length and temperature from winter to spring. Summer and autumn productivity are negatively affected by water deficit in unusually dry years, which disrupts the intrinsic seasonal growth patterns of species. The same interspecific differences in growth response observed when data were averaged across years were obtained when each year was analyzed separately. Therefore, we only present results for the spring growth response averaged across years.

### Statistical analyses

ANOVA was used to test for differences between mixtures in mean daily productivity and CWM of LES traits and for differences in morphological traits and spring growth response between species. Data for spring growth response were log‐transformed to remove differences in within‐group variance between species. Post hoc tests for significance between mixtures or species were performed using Tukey's honest significant difference. In no instance was there a significant block effect, or a significant block x treatment interaction. We used linear mixed‐effects models with block and plot as random factors to test for the effects on mean daily productivity of year, base grass species identity, and the presence of other species (chicory and plantain; timothy and prairie grass; red clover; lucerne) in sown mixtures, as well as interaction effects. Akaike's information criterion (AIC) was used to choose the most parsimonious model (i.e., the model giving the best fit to the data with the fewest fitted parameters). Tukey's honest significant difference was used to test for significant differences between species combinations within years in the final model. These analyses could not separate the effects of chicory and plantain or timothy and prairie grass because they were always sown in the same mixtures. Also, the effect of white clover could not be tested because it was sown in all mixtures. Post hoc tests for significance between pairs of groups were performed using Tukey's honest significant difference. In all analyses, there was no evidence of an interaction between block and main effects.

Jack‐knifed (“leave‐one‐out”) linear regression (Tukey 1958) was used to test for significant relationships between CWM and mean daily productivity. This reduces the possibility of obtaining a significant relationship driven by only one or two extreme values. To explore the effect of base grass species identity on trait–productivity relationships separate regression analyses were performed for plots containing either ryegrass or tall fescue as the base grass.

## Results

### Effect of mixture type and individual species on productivity

**Figure 1:**
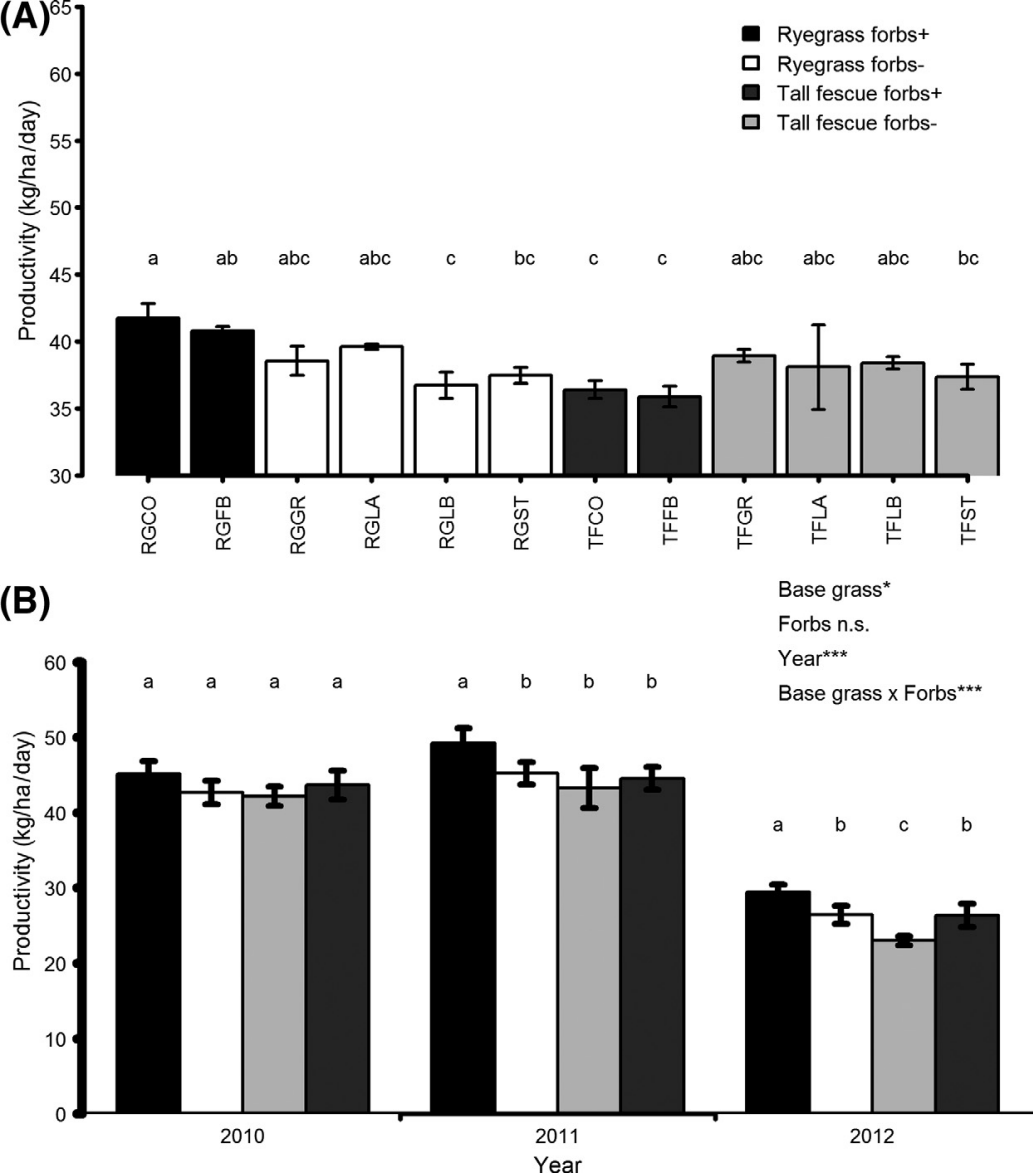
Mean aboveground productivity of (A) plots for individual mixtures averaged over the 3 years of the trial and (B) plots based on perennial ryegrass (*Lolium perenne,* RG) or tall fescue (*Festuca arundinacea*, TF) either with or without the forbs plantain (*Plantago lanceolata*) and chicory (*Chicorium intybus*) in each year of the trial. Error bars show the standard error. Letters assigned to mixtures indicate significant differences using Tukey's honest significant difference, with mixtures not sharing any letters being significantly different from each other. Sown species composition of each mixture code is given in Table 1.

There was a significant (*P *=* *0.035) effect of mixture type on mean productivity (Fig. 1). Post hoc tests revealed that the ryegrass complex (RGCO) mixture had significantly higher productivity than the ryegrass standard (RGST), ryegrass standard with lucerne (RGLB), tall fescue standard with forbs (TFFB), and tall fescue complex (TFCO) mixtures. There was no evidence for significant main effects or interactions involving the additional legumes red clover and lucerne, or the additional grasses timothy and prairie grass on productivity. Base grass identity and year had significant effects on productivity (*P *<* *0.05 and *P *<* *0.001 respectively, Fig. 1) and the interaction between base grass identity and the presence of the two forb species (chicory and plantain) was strongly significant (*P *<* *0.001). There was no evidence for any two‐way interactions involving year nor was there any for the three‐way interaction (Table S2a). Indeed, multimodel comparisons showed that the model including main effects for Year, Base grass identity and Forbs, and the interaction between Base grass identity and Forbs had very strong AIC weight support (AIC weight = 0.958, giving a 95.8% chance of providing the most parsimonious fit to the data, Table S2b) among models including all possible combinations of the three predictors and their interactions. Post hoc tests revealed that ryegrass‐based mixtures including forbs had significantly higher productivity than all other treatment combinations in the second and third years of the experiment (2011 and 2012), but not the first year (2010), although it was still the most productive combination in this year. By contrast, tall fescue mixtures including forbs had significantly lower productivity than tall fescue mixtures without forbs in 2012, but not 2010 or 2011, although in all years these mixtures had the lowest productivity of all base grass x forb combination. These results show that the effect of adding forb species to pasture mixtures is highly dependent on the identity of the grass species on which the mixture is based, and that the contrasting effects of forbs on either base grass are fairly consistent across years.

### Leaf economics spectrum traits of sown species

**Figure 2:**
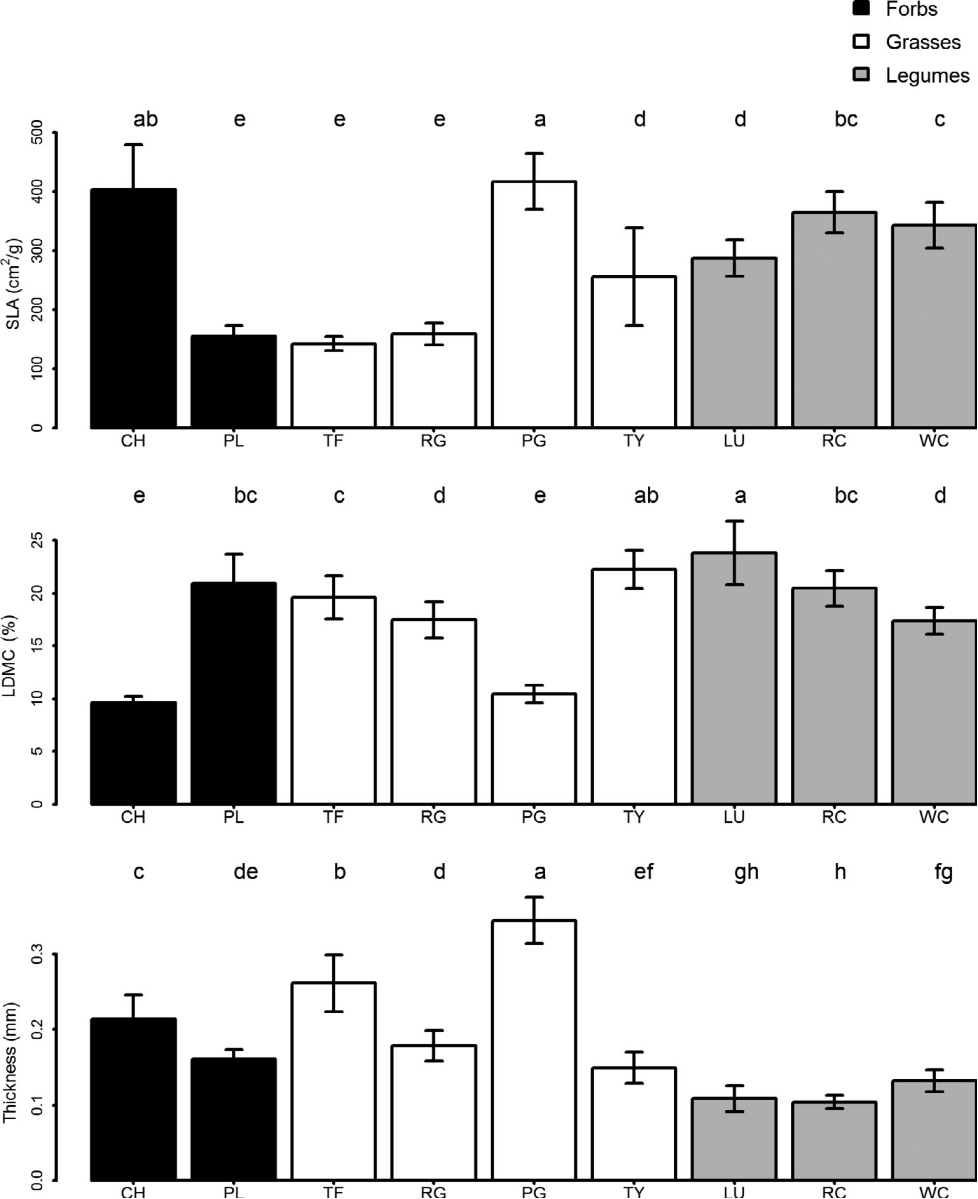
Species mean leaf trait values. Letters at the top of each subfigure indicate significant differences between species using Tukey's honest significant difference, where species that do not share any letters are significantly different from each other. “SLA” is specific leaf area, “LDMC” is leaf dry matter content, and “Thickness” is leaf thickness. Species codes are as follows: CH, chicory; LU, lucerne; PG, prairie grass; PL, plantain; RC, red clover; RG, ryegrass; TF, tall fescue; TY, timothy; WC, white clover. Scientific names and cultivars sown for each species are given in the methods section.

Three of the grasses – ryegrass, tall fescue, and prairie grass – had the lowest SLA values (Fig. 2). This was due to moderate to high (although not extreme) values for both leaf thickness and LDMC. Chicory, plantain, red clover, and white clover, had the highest SLA values. Lucerne had significantly lower SLA than both forb species and both clover species. The two forb species had the 1st and 3rd highest leaf thickness values and lowest LDMC values of any species (Fig. 2). Lucerne had the lowest leaf thickness and the highest LDMC values. Red clover had the lowest leaf thickness of all species. It also had significantly higher LDMC than white clover, ryegrass, and the forb species.

### Effect of trait composition on productivity

**Figure 3:**
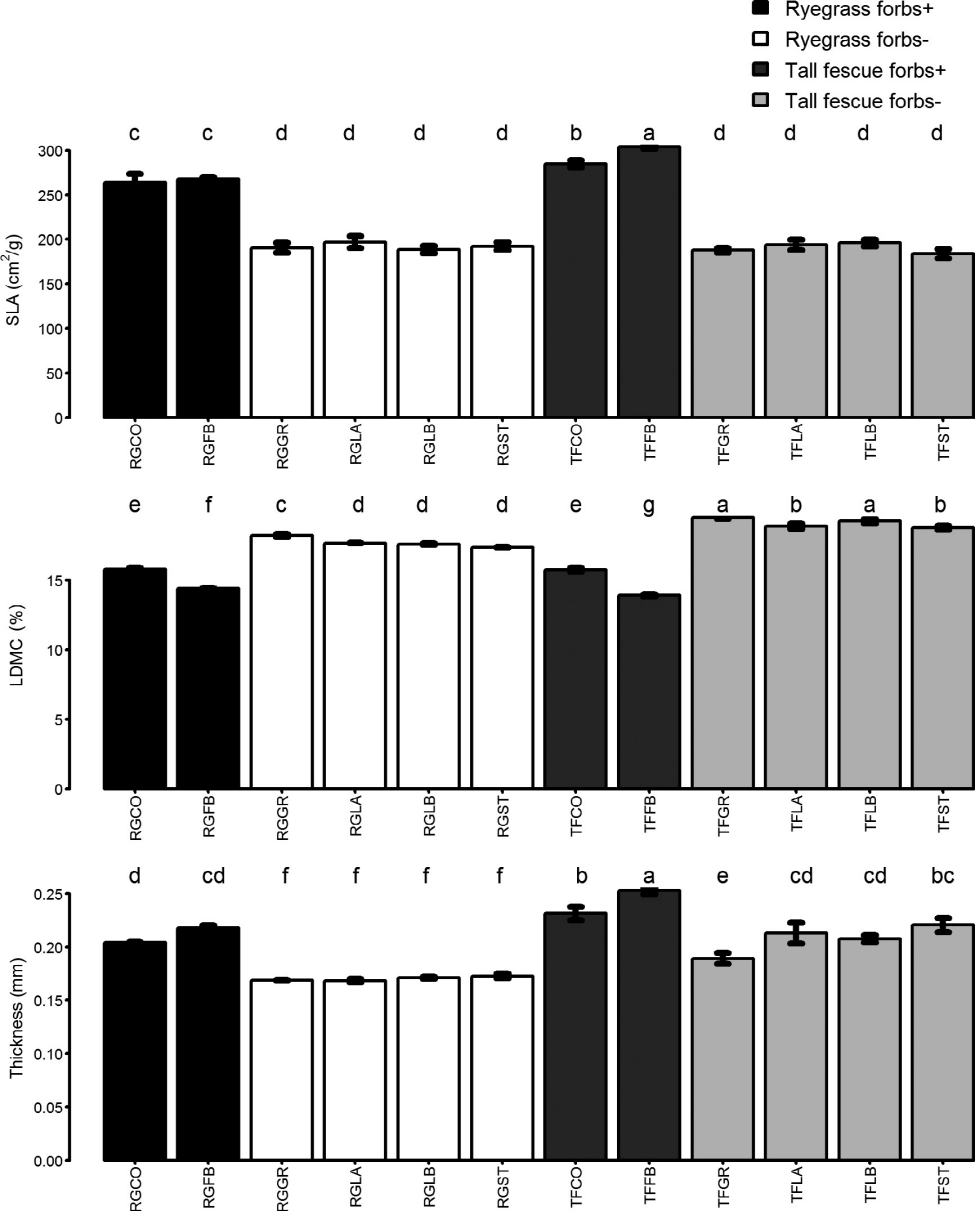
Mean biomass‐weighted trait values for each mixture (taken across plots). Letters at the top of each subfigure indicate significant differences between mixtures using Tukey's honest significant difference, where mixtures that do not share any letters are significantly different from each other. “SLA” is specific leaf area, “LDMC” is leaf dry matter content, and “Thickness” is leaf thickness. Sown species composition of each mixture code is given in Table 1.

The two most productive mixtures (RGCO and RGFB) had very similar CWM values to the two least productive mixtures (TFCO and TFFB) for SLA and LDMC (Fig. 3). All these mixtures had much higher SLA and lower LDMC than all of the other mixtures. These mixtures were also among those with the highest CWM values for leaf thickness. The patterns for CWM values presented in Figure 3 were driven by the presence of forb species. The percentage of total biomass contributed by the forbs (measured as their percentage contribution to total dry matter yield over the entire experiment) differed between mixtures with different base grasses, but they accounted for a non‐negligible percentage of total biomass whenever they were sown (36% in ryegrass‐based mixtures and 53% in tall fescue‐based mixtures). The relative abundance of forbs varied considerably between years (Fig. S2), peaking in the second year for both ryegrass‐ and tall fescue‐based mixtures. However, their abundance was always greater in tall fescue‐based mixtures.

**Figure 4:**
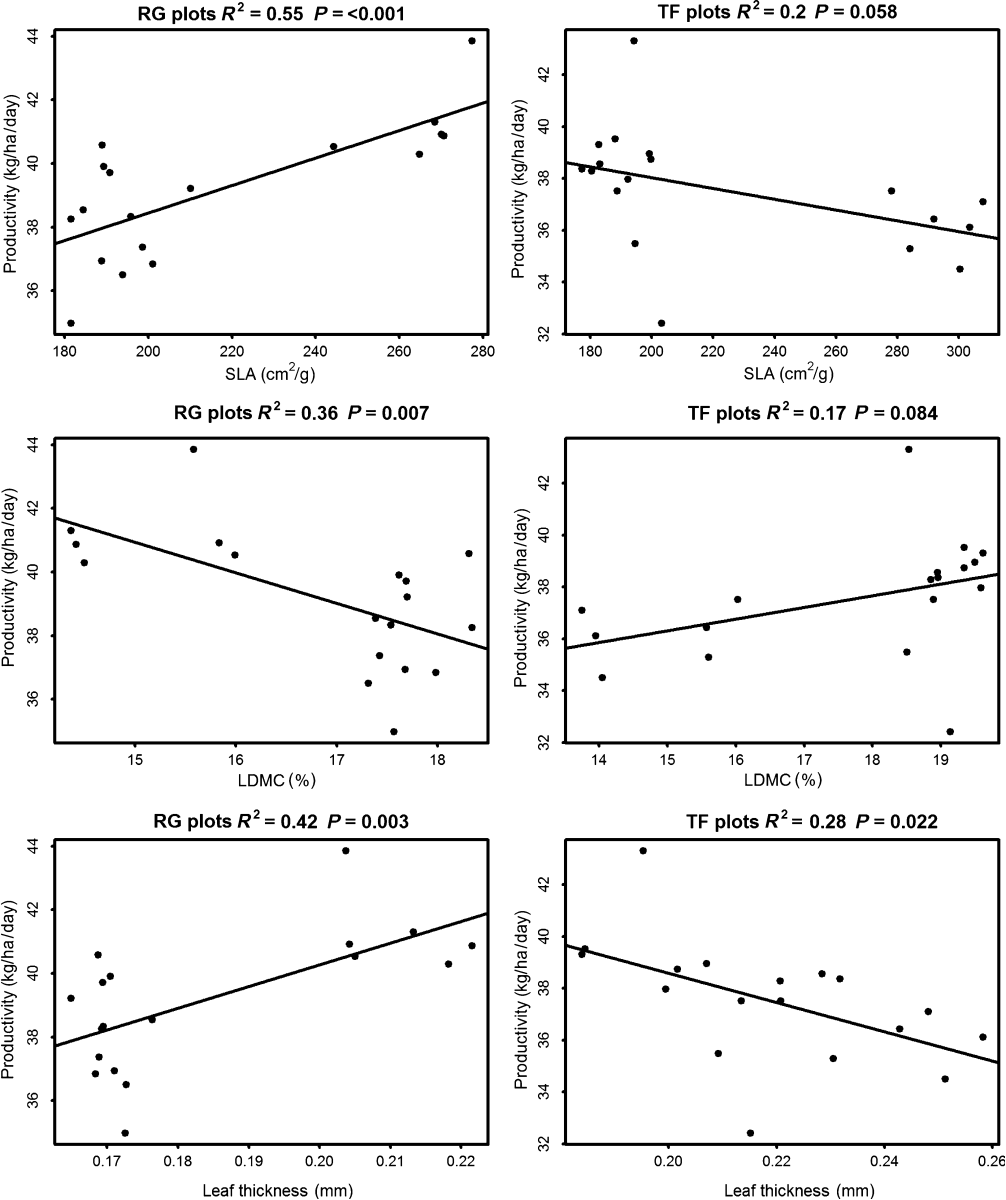
Relationships between productivity and biomass‐weighted trait values for plot based on perennial ryegrass (RG plots) or by tall fescue (TF plots). R‐square and *P* values are from jack‐knifed linear regression. “SLA” is specific leaf area and “LDMC” is leaf dry matter content.

Overall, the comparison of CWM values across all mixtures suggests LES traits do not explain differences in productivity between mixtures. This was supported by jack‐knifed regression analyses, where no significant relationships were found between plot CWM values and mean productivity (Fig. S3 in Supplementary Material). However, when ryegrass‐ and tall fescue‐based mixtures were considered separately, contrasting relationships between traits and productivity were obtained. For ryegrass‐based mixtures, productivity increased significantly with CWM for SLA and leaf thickness, and declined significantly with CWM for LDMC (Fig. 4). For tall fescue‐based mixtures, there was a nonsignificant positive trend (*P *<* *0.1) between productivity and CWM for LDMC (Fig. 4). There was also a nonsignificant (*P *<* *0.1) negative trend between leaf thickness and productivity and a significant negative relationship (*P *=* *0.022) between productivity and CWM for SLA and productivity. FDiv was not positively related to productivity for any of the traits across ryegrass‐ or tall fescue‐based mixtures (Fig. S4).

There was a strong colinearity between CWM values for different traits across the ryegrass‐based plots (Table S3). This makes it difficult to determine which metrics of functional trait structure most strongly influenced productivity. Further, as the observed relationships seem largely to be driven by the inclusion of forb species in ryegrass mixtures, there is a possibility that some other aspect of their function drives the patterns observed for the traits we measured.

### Phenological complementarity and competition between grasses and forbs

**Figure 5:**
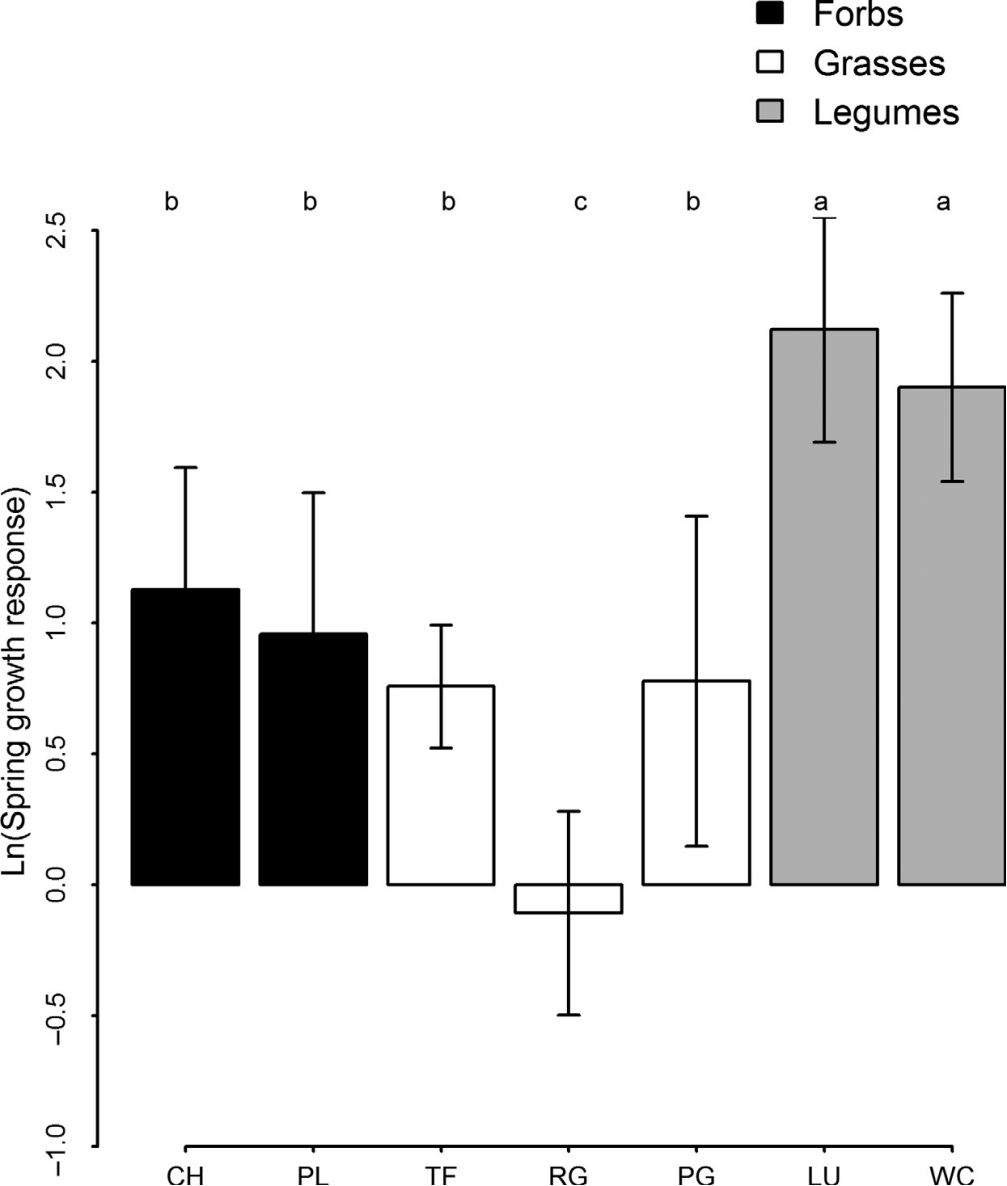
Log of “spring growth response” (i.e., difference between winter and spring growth as a proportion of winter growth) for each species. Each point is the mean value (taken across years) for each plot that a species was sown in. Values greater than zero indicate that spring growth was more than double winter growth. Letters at the top of each subfigure indicate significant differences between species using Tukey's honest significant difference, where species that do not share any letters are significantly different from each other. Species codes are as follows: CH, chicory; LU, Lucerne; PL, plantain; PG, prairie grass; RG, ryegrass; TF, tall fescue; WC, white clover. Scientific names and cultivars sown for each species are given in the methods section. For two species – red clover and timothy – there were too many missing values to obtain a reliable estimate of spring growth response.

Base grass species differed markedly in how their contribution to productivity responded to the presence of forbs. Ryegrass contribution to productivity was 25% lower in mixtures including forbs while the tall fescue contribution to productivity was 51% lower in mixtures containing forbs. This suggests that the competitive effect of forb species on tall fescue was twice as strong as that for ryegrass. There were significant differences between species in spring growth response (Fig. 5). Post hoc tests showed that ryegrass had a significantly lower response than all other species, meaning that its growth was comparatively consistent across seasons. Indeed, ryegrass maintained a relatively high level of productivity in winter, with more than 20% of its total annual dry matter production occurring in this season, compared with 13% for tall fescue, 14% for plantain, and 7% for chicory. Lucerne and white clover had a significantly higher spring growth response than all other species (Fig. 5). There was no evidence for differences between either of the forb species and tall fescue or prairie grass, but they had values intermediate between those of ryegrass or lucerne and white clover. This suggests the seasonal growth patterns of forb species differed markedly from ryegrass but not from tall fescue.

Seasonal patterns in plot‐level productivity confirm the phenological differences between base grasses. Mixtures based on ryegrass had much higher winter productivity than those which did not, while mixtures based on tall fescue had significantly higher spring productivity (Fig. S5). Further, examination of seasonal productivity for the two base grasses and forbs shows that ryegrass experiences a much smaller proportional increase in productivity from each winter to the following spring and a much smaller decline in yield from each autumn to the following winter than either the forbs or tall fescue (Fig. S6). Taken together with the findings for the spring growth response index, and the relative effects of forbs on either base grass, these results suggest phenological complementarity reduced competition between forbs and ryegrass relative to competition between forbs and tall fescue. This in turn may explain why forbs enhanced productivity in ryegrass‐based mixtures, but had a negative effect in tall fescue‐based mixtures.

## Discussion

The results show that dominant species identity can strongly influence leaf economics spectrum (LES) trait–productivity relationships. For ryegrass‐based mixtures, strong significant relationships were found between productivity and functional composition (community‐weighted mean, CWM) for key LES traits (SLA, LDMC, and leaf thickness). For tall fescue‐based mixtures, no such relationship was observed. This was due to the contrasting effects on productivity of species with leaf traits linked to rapid photosynthesis on productivity (the forbs chicory and plantain) in mixtures based on either grass species.

Forb effects on productivity appeared to be contingent on their phenological complementarity with the base grass species. Forbs had a net negative or neutral impact on productivity when they were phenologically similar to the base grass. When they differed in phenology from the base grass, forbs appeared to have a net positive effect on productivity. Forbs seemed to compete much more strongly with phenologically similar tall fescue. They caused greater decreases in its contribution to total productivity than for the phenologically different ryegrass. This apparently nullified or outweighed any positive effects forbs might have had on productivity when grown in mixture with tall fescue. The lower competitive effect of forbs on ryegrass could be due reductions in both aboveground and belowground competition. Available evidence suggests that tall fescue not only has much lower aboveground biomass in winter than ryegrass, but lower belowground (nitrogen) resource uptake as well (Malcolm et al. 2014). If the same is true for the winter‐dormant forb species then their phenological differences with ryegrass could reduce competition for belowground resources.

Care is required in drawing generalizations from the results because of the small pool of species involved and that only a single cultivar of each species was used. Nevertheless, the results raise some interesting questions for LES trait–productivity relationships in general, and for intensively managed pastoral systems in particular Specifically, they hint that phenological complementarity, combined with species having traits linked to high RGR, could increase agricultural productivity without increasing inputs. This could have considerable benefits for enhancing the sustainability of food‐producing systems (Tilman et al. 2002).

### Trait–productivity relationships and species interactions

There is a strong theoretical and empirical basis for expecting that LES traits will generally influence ecosystem‐level productivity via the mass ratio hypothesis (Grime 1998; Shipley 2006; Vile et al. 2006). However, our findings show that when species with LES traits, which in theory should enhance function, compete intensely with other species, their net effect on total productivity may be neutral or even negative. In such instances, there will be no relationship between LES trait values and productivity. It seems LES traits may only be related to productivity when complementarity in other trait axes, such as phenology, reduces interspecific competition.

This supports the idea that both the mass ratio hypothesis and complementarity are likely to influence productivity in most plant communities (Mouillot et al. 2011), but that they will do so for different types of traits. The results suggest increased abundance of fast‐growing species and phenological complementarity are both necessary for increasing productivity. Other trait axes linked to resource acquisition (i.e., when and where plants obtain their resources from) could also influence productivity via complementarity. For instance, inclusion of N‐fixers in mixtures generally enhances productivity via differentiation in N resources (Petchey et al. 2004; Roscher et al. 2012; Finn et al. 2013; Suter et al. 2015). Similarly, differences in rooting depth can reduce competition and promote co‐existence between species (Fargione and Tilman 2005). It would be very interesting to have more experiments to test whether the mechanism by which traits influence productivity (i.e., mass ratio or complementarity) depends on whether a trait is linked to either RGR or resource acquisition.

The findings are perhaps surprising in suggesting that complementarity may have a strong positive effect on productivity even in systems subjected to frequent disturbance, where the effects of competition are expected to be less obvious (Grime 2001). However, there is an increasing recognition in the agricultural literature that building seasonal complementarity into intensive pastoral farming systems can greatly increase yields without increasing environmental impacts (Tow et al. 1997; Moore et al. 2004; Garcia et al. 2008; Rawnsley et al. 2013). Evidence is also emerging that complementarity in establishment rate might benefit productivity in intensive pastures over multiple years. For instance, Finn et al. (2013), in a 3‐year study across 30 sites spanning almost the entire latitudinal range of Europe, demonstrated over‐yielding (sensu Loreau and Hector 2001) in mixtures including fast‐establishing and perennial persistent grasses and legumes increased productivity.

### How important is species composition for productivity in intensive systems?

Pasture mixtures that increase resource‐use complementarity and include species with traits linked to very rapid photosynthetic rates could have material benefits for productivity in intensive agricultural systems. Adding forbs to ryegrass‐based mixtures increased productivity by more than 1.3 t·ha^−1^·year^−1^ in this study. To put this in perspective, a 10‐year trial on the same farm we used showed that the average increase in production from adding nitrogen fertilizer (at a rate of 180 kg·N·ha^−1^·year^−1^) was 2.9 t·ha^−1^·year^−1^ (Glassey et al. 2013). Thus, the benefit of adding forbs is almost 50% of that derived from fertilizer addition. This emphasizes the potential for new pasture mixtures to increase productivity without increasing inputs, or for maintaining productivity while reducing inputs. This in turn could have great benefits for attempts to maintain productivity within environmental limits, by reducing nutrient inputs and hence leaching to waterways, while also increasing farm profitability by reducing fertilizer costs and reliance on imported feed.

As noted above, caution is necessary in generalizing the results of this study because of the small species pool used. Generality of the findings needs to be tested on different sets of species. However, this may be somewhat challenging, given the small global species pool available for intensively managed pasture mixtures. Indeed, Finn et al. (2013) used a total of only nine species in their pan‐European study. Another option may be to make more detailed measurements of species function, such as leaf‐level photosynthesis and relative growth rate, to confirm that the leaf traits of forb species do allow them to grow more rapidly. It might also be useful to measure net ecosystem carbon exchange (Milcu et al. 2014), to confirm that including forbs in mixtures increases peak carbon sequestration, predictable based on their leaf traits. Pairwise competition experiments could confirm that the forbs really do compete more strongly with tall fescue than ryegrass (Wilson and Roxburgh 2001). Finally, to test properly whether forbs enhance over‐yielding through complementarity would require an experiment including monocultures and ryegrass‐based mixtures with and without forbs (Loreau and Hector 2001).

## Conclusions

The results of this study show that competitive interactions strongly modify the influence of LES traits on productivity. Productivity was maximized when fast‐growing forb species competed less intensely with the dominant grass species. The study also suggests that the mass ratio and complementarity hypotheses are both likely to influence productivity, but for different traits. The mass ratio hypothesis should dominate for traits linked to maximum relative growth rate, while complementarity effects should be strongest for traits linked to spatial or temporal resource‐use differentiation between species. Finally, it has been shown that novel species combinations can enhance agricultural production for the same level of inputs, which could have huge benefits for enhancing the sustainability of our food‐producing systems.

## Conflict of Interest

None declared.

## Supporting information


**Figure S1**. Allocation of treatments to plots in the experiments.

**Figure S2.** Mean combined abundance of the forbs chicory and plantain in ryegrass and tall fescue‐based plots (where sown) for each of the three years of the experiment.

**Figure S3.** Relationships between productivity and biomass‐weighted trait values for all plots.

**Figure S4.** Relationships between productivity and functional divergence (FDiv) across either ryegrass or tall fescue‐based plots (RG plots and TG plots respectively).

**Figure S5.** Productivity of ryegrass‐based and non‐ryegrass‐based plots in different seasons.

**Figure S6.** Mean biomass yield of the forb species (chicory and plantain), tall fescue and ryegrass in each season in each year.

**Table S1.** Seed sowing rates for each species in each mixture.

**Table S2.** ANOVA table for a linear mixed‐effects model (A) including Year, Base grass identity, presence or absence of forbs and all possible interactions between them as fixed effects, with plot and block as random effects; and results from multimodel comparisons for all possible combinations of the predictors and their interactions (B).

**Table S3.** Pearson correlations between biomass‐weighted trait values across ryegrass‐dominated plots.

## References

[ece31964-bib-0001] Clark, C. M. , D. F. B. Flynn , B. J. Butterfield , and P. B. Reich . 2012 Testing the link between functional diversity and ecosystem functioning in a Minnesota grassland experiment. PLoS ONE, 7:e52821. 23300787 10.1371/journal.pone.0052821 PMC3534119

[ece31964-bib-0002] Fargione, J. , and D. Tilman . 2005 Niche differences in phenology and rooting depth promote coexistence with a dominant C‐4 bunchgrass. Oecologia 143:598–606. 15791430 10.1007/s00442-005-0010-y

[ece31964-bib-0003] Finn, J. A. , L. Kirwan , J. Connolly , M. T. Sebastia , A. Helgadottir , O. H. Baadshaug , et al. 2013 Ecosystem function enhanced by combining four functional types of plant species in intensively managed grassland mixtures: a 3‐year continental‐scale field experiment. J. Appl. Ecol. 50:365–375.

[ece31964-bib-0004] Garcia, S. C. , W. J. Fulkerson , and S. U. Brookes . 2008 Dry matter production, nutritive value and efficiency of nutrient utilization of a complementary forage rotation compared to a grass pasture system. Grass Forage Sci. 63:284–300.

[ece31964-bib-0005] Garnier, E. , J. Cortez , G. Billès , M.‐L. Navas , C. Roumet , M. Debussche , et al. 2004 Plant functional markers capture ecosystem properties during secondary succession. Ecology 85:2630–2637.

[ece31964-bib-0006] Glassey, C. B. , C. G. Roach , J. M. Lee , and D. A. Clark . 2013 The impact of farming without nitrogen fertiliser for ten years on pasture yield and composition, milksolids production and profitability; a research farmlet comparison. Proc. NZ Grassl. Assoc. 75:71–78.

[ece31964-bib-0007] Grime, J. P. 1998 Benefits of plant diversity to ecosystems: immediate, filter and founder effects. J. Ecol. 86:902–910.

[ece31964-bib-0008] Grime, J. P. 2001 Plant strategies, vegetation processes, and ecosystem properties, 2 edn Wiley, Chichester, UK.

[ece31964-bib-0588] Hewitt, A. E. 1993 New Zealand Soil Classification. Manaaki Whenua ‐ Landcare Research Ltd, Lincoln.

[ece31964-bib-0009] Lepš, J. 2004 What do the biodiversity experiments tell us about consequences of plant species loss in the real world? Basic Appl. Ecol. 5:529–534.

[ece31964-bib-0010] Loreau, M. , and A. Hector . 2001 Partitioning selection and complementarity in biodiversity experiments. Nature 412:72–76. 11452308 10.1038/35083573

[ece31964-bib-0011] Malcolm, B. J. , K. C. Cameron , H. J. Di , G. R. Edwards , and J. L. Moir . 2014 The effect of four different pasture species compositions on nitrate leaching losses under high N loading. Soil Use Manag. 30:58–68.

[ece31964-bib-0012] Mason, N. W. H. , N. Pipenbaher , S. Škornik , and M. Kaligarič . 2013 Does complementarity in leaf phenology and inclination promote co‐existence in a species‐rich meadow? Evidence from functional groups. J. Veg. Sci. 24:94–100.

[ece31964-bib-0013] Milcu, A. , C. Roscher , A. Gessler , D. Bachmann , A. Gockele , M. Guderle , et al. 2014 Functional diversity of leaf nitrogen concentrations drives grassland carbon fluxes. Ecol. Lett. 17:435–444. 24393400 10.1111/ele.12243

[ece31964-bib-0014] Mokany, K. , J. Ash , and S. Roxburgh . 2008 Functional identity is more important than diversity in influencing ecosystem processes in a temperate native grassland. J. Ecol. 96:884–893.

[ece31964-bib-0015] Moore, K. J. , T. A. White , R. L. Hintz , P. K. Patrick , and E. C. Brummer . 2004 Sequential grazing of cool‐ and warm‐season pastures. Agron. J. 96:1103–1111.

[ece31964-bib-0016] Mouchet, M. A. , S. Villeger , N. W. H. Mason , and D. Mouillot . 2010 Functional diversity measures: an overview of their redundancy and their ability to discriminate community assembly rules. Funct. Ecol. 24:867–876.

[ece31964-bib-0017] Mouillot, D. , S. Villeger , M. Scherer‐Lorenzen , and N. W. H. Mason . 2011 Functional structure of biological communities predicts ecosystem multifunctionality. PLoS ONE, 6:e17476. 21423747 10.1371/journal.pone.0017476 PMC3053366

[ece31964-bib-0018] Niinemets, U. 1999 Components of leaf dry mass per area – thickness and density ‐ alter leaf photosynthetic capacity in reverse directions in woody plants. New Phytol. 144:35–47.

[ece31964-bib-0019] Perez‐Harguindeguy, N. , S. Diaz , E. Garnier , S. Lavorel , H. Poorter , P. Jaureguiberry , et al. 2013 New handbook for standardised measurement of plant functional traits worldwide. Aust. J. Bot. 61:167–234.

[ece31964-bib-0020] Petchey, O. L. , A. Hector , and K. J. Gaston . 2004 How to different measures of functional diversity perform? Ecology 85:847–857.

[ece31964-bib-0021] Quested, H. , O. Eriksson , C. Fortunel , and E. Garnier . 2007 Plant traits relate to whole‐community litter quality and decomposition following land use change. Funct. Ecol. 21:1016–1026.

[ece31964-bib-0022] Rawnsley, R. P. , D. F. Chapman , J. L. Jacobs , S. C. Garcia , M. N. Callow , G. R. Edwards , et al. 2013 Complementary forages – integration at a whole‐farm level. Anim. Prod. Sci. 53:976–987.

[ece31964-bib-0023] Reich, P. B. , D. S. Ellsworth , M. B. Walters , J. M. Vose , C. Gresham , J. C. Volin , et al. 1999 Generality of leaf trait relationships: a test across six biomes. Ecology 80:1955–1969.

[ece31964-bib-0024] Roscher, C. , M. Scherer‐Lorenzen , J. Schumacher , V. M. Temperton , N. Buchmann , and E. D. Schulze . 2011 Plant resource‐use characteristics as predictors for species contribution to community biomass in experimental grasslands. Perspect. Plant Ecol. Evol. Syst. 13:1–13.

[ece31964-bib-0025] Roscher, C. , J. Schumacher , M. Gubsch , A. Lipowsky , A. Weigelt , N. Buchmann , et al. 2012 Using plant functional traits to explain diversity‐productivity relationships. PLoS ONE, 7:e36760. 22623961 10.1371/journal.pone.0036760 PMC3356333

[ece31964-bib-0026] Shipley, B. 2006 Net assimilation rate, specific leaf area and leaf mass ratio: which is most closely correlated with relative growth rate? A meta‐analysis. Funct. Ecol. 20:565–574.

[ece31964-bib-0027] Stevens, M. H. H. , and W. P. Carson . 2001 Phenological complementarity, species diversity, and ecosystem function. Oikos 92:291–296.

[ece31964-bib-0028] Storkey, J. , T. Döring , J. Baddeley , R. Collins , S. Roderick , H. Jones , et al. 2015 Engineering a plant community to deliver multiple ecosystem services. Ecol. Appl. 25:1034–1043. 26465040 10.1890/14-1605.1

[ece31964-bib-0029] Suter, M. , J. Connolly , J. A. Finn , R. Loges , L. Kirwan , M.‐T. Sebastià , et al. 2015 Nitrogen yield advantage from grass–legume mixtures is robust over a wide range of legume proportions and environmental conditions. Glob. Change Biol. 21:2424–2438. 10.1111/gcb.12880 25626994

[ece31964-bib-0030] Tilman, D. , J. Knops , D. Wedin , P. Reich , M. Ritchie , and E. Siemann . 1997 The influence of functional diversity and composition on ecosystem processes. Science 277:1300–1302.

[ece31964-bib-0031] Tilman, D. , K. G. Cassman , P. A. Matson , R. Naylor , and S. Polasky . 2002 Agricultural sustainability and intensive production practices. Nature 418:671–677. 12167873 10.1038/nature01014

[ece31964-bib-0032] Tow, P. G. , J. V. Lovett , and A. Lazenby . 1997 Adaptation and complementarity of *Digitaria eriantha* and *Medicago sativa* on a solodic soil in a subhumid environment with summer and winter rainfall. Aust. J. Exp. Agric. 37:311–322.

[ece31964-bib-0033] Tukey, J. W. 1958 Bias and confidence in not‐quite large samples. Ann. Math. Stat. 29:614.

[ece31964-bib-0034] Vile, D. , B. Shipley , and E. Garnier . 2006 Ecosystem productivity can be predicted from potential relative growth rate and species abundance. Ecol. Lett. 9:1061–1067. 16925655 10.1111/j.1461-0248.2006.00958.x

[ece31964-bib-0035] Villeger, S. , N. W. H. Mason , and D. Mouillot . 2008 New multidimensional functional diversity indices for a multifaceted framework in functional ecology. Ecology 89:2290–2301. 18724739 10.1890/07-1206.1

[ece31964-bib-0036] Wilson, J. B. , and S. H. Roxburgh . 2001 Intrinsic guild structure: determination from competition experiments. Oikos 92:189–192.

[ece31964-bib-0037] Wright, I. J. , P. B. Reich , M. Westoby , D. D. Ackerly , Z. Baruch , F. Bongers , et al. 2004 The worldwide leaf economics spectrum. Nature 428:821–827. 15103368 10.1038/nature02403

