# Supplementary material for: Leaf economics spectrum–productivity relationships in intensively grazed pastures depend on dominant species identity
Source: Ecol Evol. 2016 Apr 2;6(10):3079–91. doi: 10.1002/ece3.1964 (PMC4821841; doi:10.1002/ece3.1964)
Supplement: Supplementary file 1 — Figure S1. Allocation of treatments to plots in the experiments. [file ECE3-6-3079-s001.pdf]

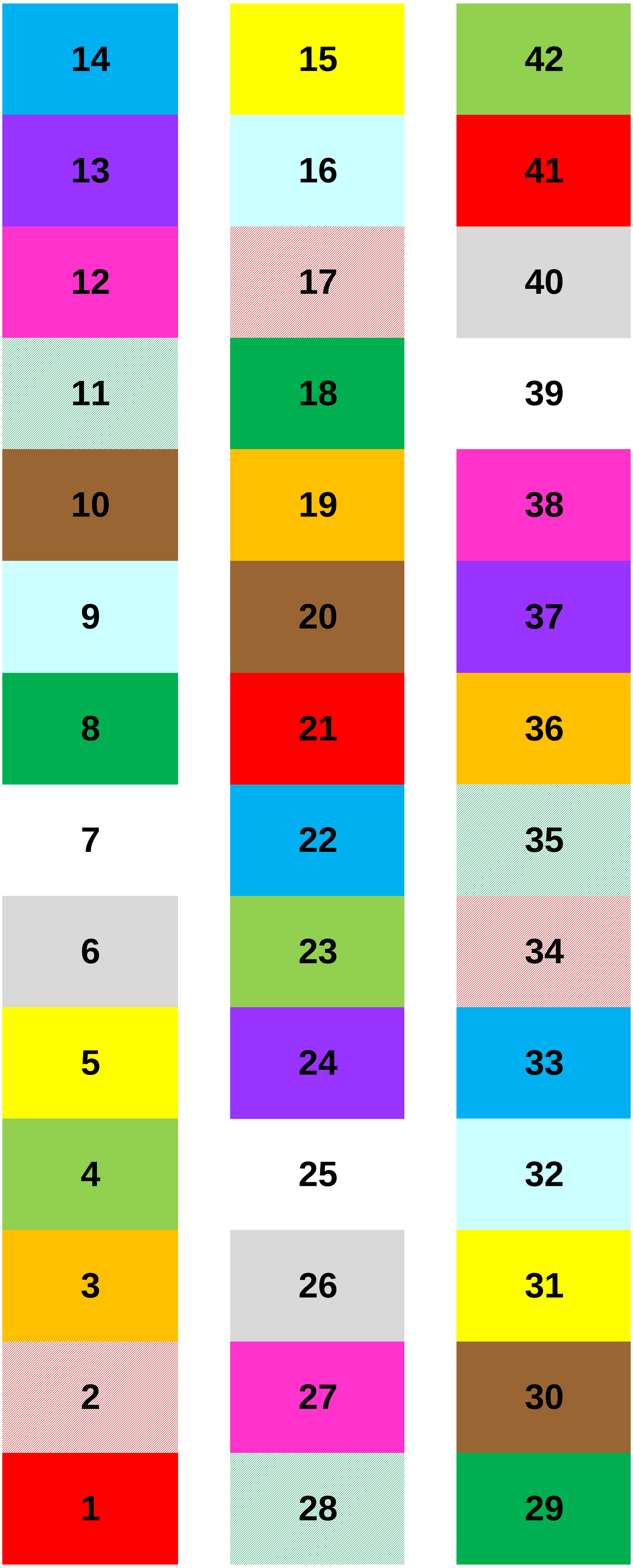

	Treatments	Abbrev
1	Ryegrass standard	**RGST**
2	Ryegrass standard + legumes A	**RGLA**
3	Ryegrass standard + legumes B	**RGLB**
4	Ryegrass standard + herbs	**RGHB**
5	Ryegrass standard + grasses	**RGGR**
6	Ryegrass complex	**RGCO**
7	Tall fescue standard	**TFST**
8	Tall fescue standard + legumes A	**TFLA**
9	Tall fescue standard + legumes B	**TFLB**
10	Tall fescue standard + herbs	**TFHE**
11	Tall fescue standard + grasses	**TFGR**
12	Tall fescue complex	**TFCO**
13	Special A	**GRLH**
14	Special B	**PGLU**
