# Supplementary figures and images for: Leaf economics spectrum–productivity relationships in intensively grazed pastures depend on dominant species identity
Source: Ecol Evol. 2016 Apr 2;6(10):3079–91. doi: 10.1002/ece3.1964 (PMC4821841; doi:10.1002/ece3.1964)

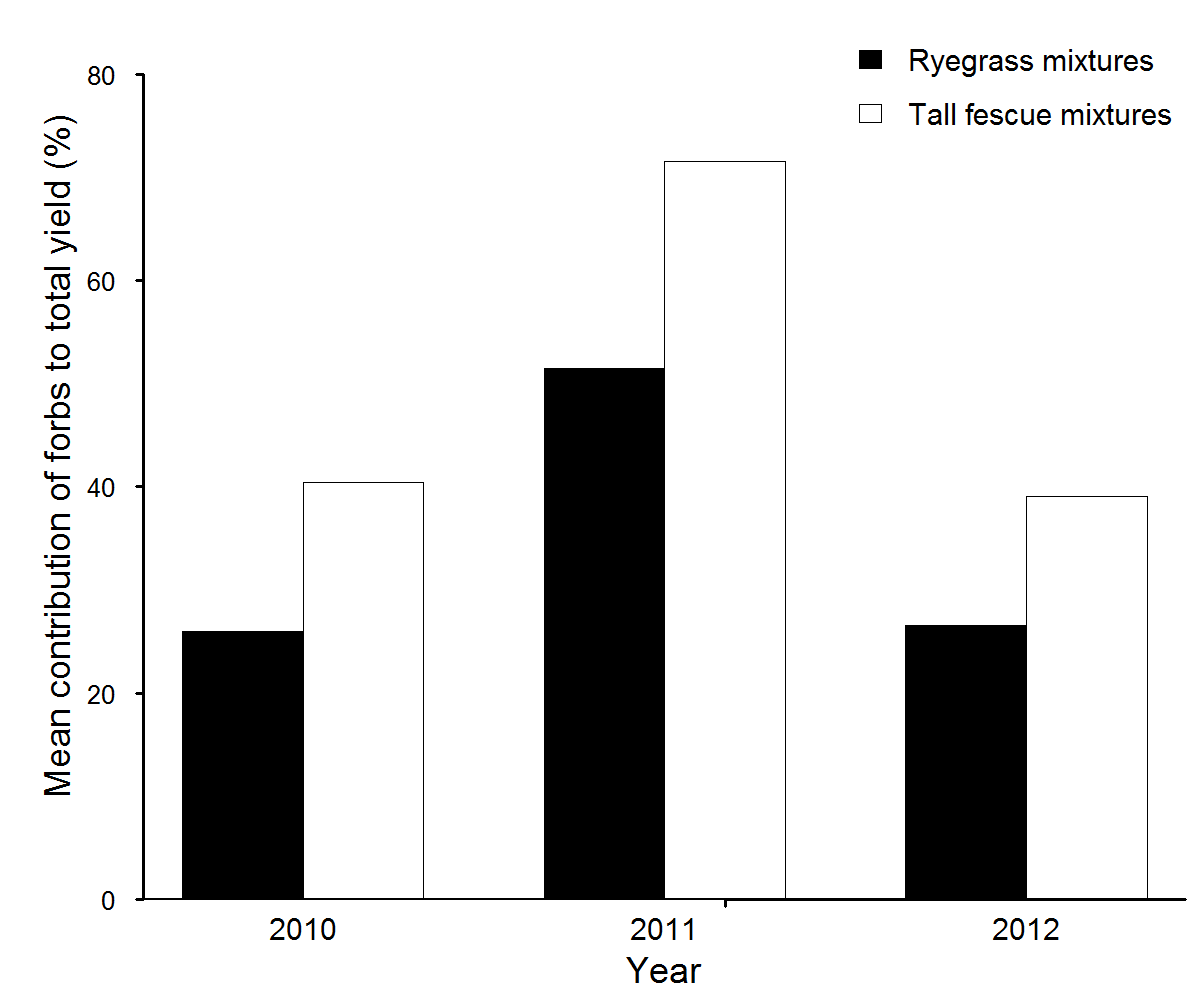

Supplement: Supplementary file 2 — Figure S2. Mean combined abundance of the forbs chicory and plantain in ryegrass and tall fescue‐based plots (where sown) for each of the three years of the experiment. [file ECE3-6-3079-s002.Tiff]

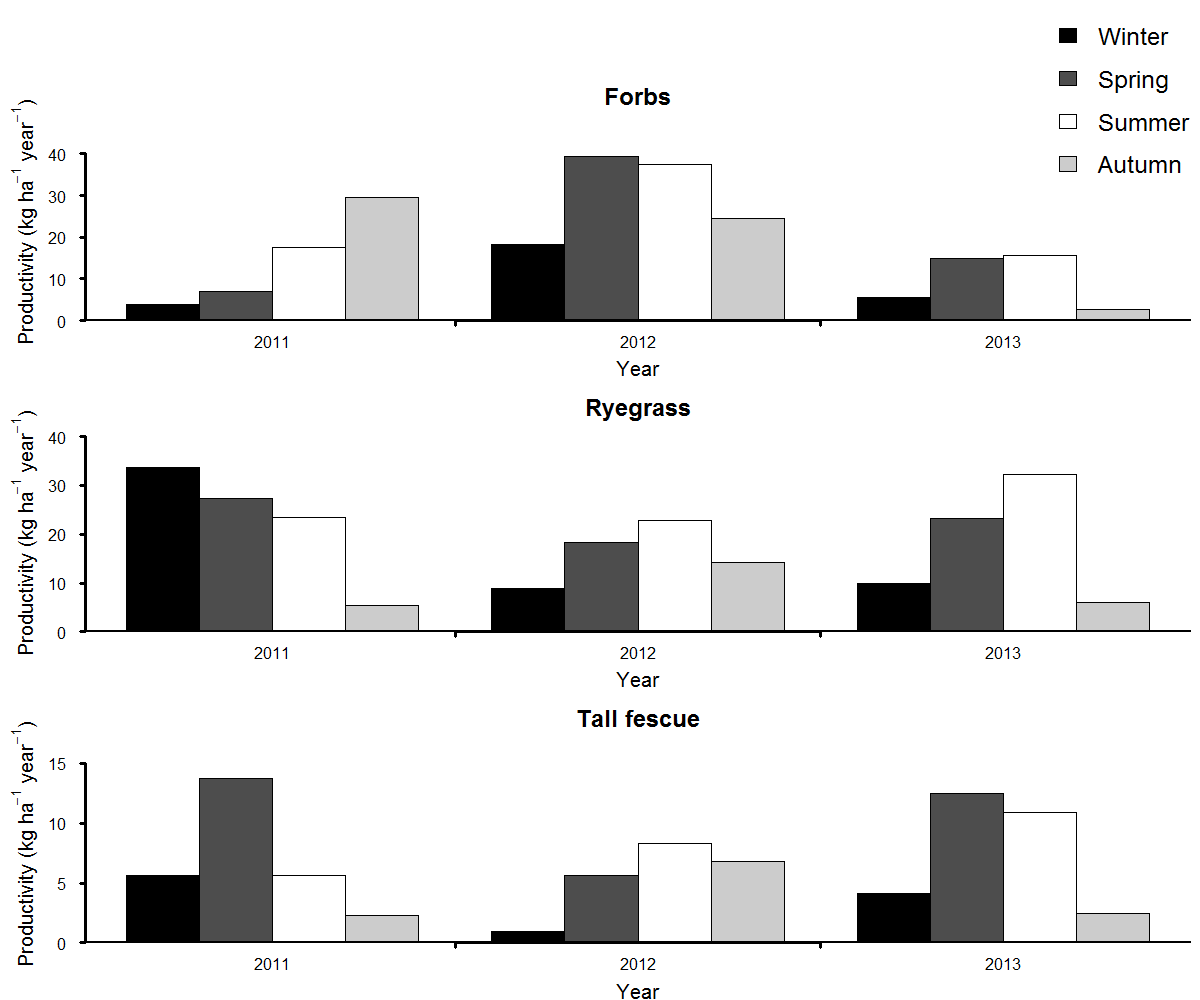

Supplement: Supplementary file 6 — Figure S6. Mean biomass yield of the forb species (chicory and plantain), tall fescue and ryegrass in each season in each year. [file ECE3-6-3079-s006.Tiff]
