# Supplementary material for: Leaf economics spectrum–productivity relationships in intensively grazed pastures depend on dominant species identity
Source: Ecol Evol. 2016 Apr 2;6(10):3079–91. doi: 10.1002/ece3.1964 (PMC4821841; doi:10.1002/ece3.1964)
Supplement: Supplementary file 7 — Table S1. Seed sowing rates for each species in each mixture. [file ECE3-6-3079-s007.docx]

**Table S1:** Seed sowing rates for each species in each mixture.

|  | **Seed rate (kg/ha)** | | | | | | | | |
| --- | --- | --- | --- | --- | --- | --- | --- | --- | --- |
| Mixture | Ryegrass | Tall fescue | White clover | Red clover | Lucerne | Plantain | Chicory | Prairie grass | Timothy |
| Ryegrass standard | 23 | . | 5 | . | . | . | . | . | . |
| Ryegrass standard + legumes A | 23 | . | 3 | 4 | . | . | . | . | . |
| Ryegrass standard + legumes B | 18 | . | 3 | . | 8 | . | . | . | . |
| Ryegrass standard + forbs | 18 | . | 3 | . | . | 1.5 | 2 | . | . |
| Ryegrass standard + grasses | 10 | . | 3 | . | . | . | . | 15 | 3 |
| Ryegrass complex | 10 | . | 1.5 | 2 | 4 | 0.75 | 1 | 15 | 3 |
| Tall fescue standard | . | 30 | 5 | . | . | . | . | . | . |
| Tall fescue standard + legumes A | . | 30 | 3 | 4 | . | . | . | . | . |
| Tall fescue standard + legumes B | . | 24 | 3 | . | 8 | . | . | . | . |
| Tall fescue standard + forbs | . | 24 | 3 | . | . | 1.5 | 2 | . | . |
| Tall fescue standard + grasses | . | 13 | 3 | . | . | . | . | 15 | 3 |
| Tall fescue complex | . | 13 | 1.5 | 2 | 4 | 0.75 | 1 | 15 | 3 |
