# Supplementary material for: Leaf economics spectrum–productivity relationships in intensively grazed pastures depend on dominant species identity
Source: Ecol Evol. 2016 Apr 2;6(10):3079–91. doi: 10.1002/ece3.1964 (PMC4821841; doi:10.1002/ece3.1964)
Supplement: Supplementary file 8 — Table S2. ANOVA table for a linear mixed‐effects model (A) including Year, Base grass identity, presence or absence of forbs and all possible interactions between them as fixed effects, with plot and block as random effects; and results from multimodel comparisons for all possible combinations of the predictors and their interactions (B). [file ECE3-6-3079-s008.docx]

**Table S2:** ANOVA table for a linear mixed effects model (A) including Year, Base grass identity, presence or absence of forbs and all possible interactions between them as fixed effects, with plot and block as random effects; and results from multi-model comparisons for all possible combinations of the predictors and their interactions (B). The AIC weight indicates the probability that the model in question provides the most parsimonious fit to the data amongst those examined.

**(A)**

| **Fixed effect** | **Df** | **Sum of squares** | **Mean square** | **F-stat** | **Sig** |
| --- | --- | --- | --- | --- | --- |
| **Year** | **2** | **7850.1** | **3925.1** | **462.9** | ******* |
| **Base** | **1** | **59.4** | **59.4** | **7.0** | ***** |
| HB | 1 | 6.5 | 6.5 | 0.8 |  |
| Year x Base | 2 | 23.2 | 11.6 | 1.4 |  |
| Year x Forbs | 2 | 9.3 | 4.7 | 0.5 |  |
| **Base x Forbs** | **1** | **133.1** | **133.1** | **15.7** | ******* |
| Year x Base x Forbs | 2 | 5.7 | 2.8 | 0.3 |  |
| Error | 114 | 966.8 | 8.5 |  |  |
|  |  |  |  |  |  |
| **(B)** |  |  |  |  |  |
| **Model** | **AIC** | **BIC** | **logLik** | **Deviance** | **AIC weight** |
| **Year + Base x Forbs** | **554.39** | **575.85** | **-269.2** | **538.39** | **0.958** |
| Year x Base x Forbs | 561.49 | 599.04 | -266.75 | 533.49 | 0.028 |
| Year + Base | 564.9 | 580.99 | -276.45 | 552.9 | 0.005 |
| Yearx Base | 565.96 | 587.41 | -274.98 | 549.96 | 0.003 |
| Year + Base + Forbs | 566.34 | 585.11 | -276.17 | 552.34 | 0.002 |
| Year x Base + Forbs | 567.39 | 591.53 | -274.7 | 549.39 | 0.001 |
| Year | 567.65 | 581.06 | -278.83 | 557.65 | 0.001 |
| Year + Forbs | 569.16 | 585.25 | -278.58 | 557.16 | 0.001 |
| Year x Forbs + Base | 569.17 | 593.31 | -275.59 | 551.17 | 0.001 |
| Year x Forbs | 572 | 593.46 | -278 | 556 | 0.000 |
| Base | 791.56 | 802.29 | -391.78 | 783.56 | 0.000 |
| Forbs | 792.34 | 803.07 | -392.17 | 784.34 | 0.000 |
| Base x Forbs | 793.46 | 809.55 | -390.73 | 781.46 | 0.000 |
| Base + Forbs | 793.46 | 806.87 | -391.73 | 783.46 | 0.000 |
