# Supplementary material for: Leaf economics spectrum–productivity relationships in intensively grazed pastures depend on dominant species identity
Source: Ecol Evol. 2016 Apr 2;6(10):3079–91. doi: 10.1002/ece3.1964 (PMC4821841; doi:10.1002/ece3.1964)
Supplement: Supplementary file 9 — Table S3. Pearson correlations between biomass‐weighted trait values across ryegrass‐dominated plots. [file ECE3-6-3079-s009.docx]

**Table S3:** Pearson correlations between biomass-weighted trait values across ryegrass-dominated plots.

|  | *LDMC* | *Leaf thickness* |
| --- | --- | --- |
| SLA | –0.926 | 0.933 |
| LDMC |  | –0.972 |
